# Placental histology, perioperative brain development, and neurodevelopmental outcome at 1 year of age in patients undergoing neonatal cardiac surgery—is there an association?

**DOI:** 10.3389/fcvm.2025.1556289

**Published:** 2025-05-30

**Authors:** Céline Steger, Alexander Boegeholz, Beatrice Latal, Maria Feldmann, Raimund Kottke, Cornelia Hagmann, Tanja Restin, Ruth Tuura O'Gorman, Andras Jakab, Michael Reinehr, Walter Knirsch

**Affiliations:** ^1^ Children's Research Center, University Children's Hospital Zurich, Zurich, Switzerland; ^2^ University of Zurich, Zurich, Switzerland; ^3^ MR Center, University Children's Hospital Zurich, Zurich, Switzerland; ^4^ Pediatric Cardiology, Pediatric Heart Center, University Children's Hospital Zurich, Zurich, Switzerland; ^5^ Institute of Pathology and Molecular Pathology, University Hospital Zurich, Zurich, Switzerland; ^6^ Child Development Center, University Children's Hospital Zurich, Zurich, Switzerland; ^7^ Neonatology and Pediatric Intensive Care, University Children's Hospital Zurich, Zurich, Switzerland; ^8^ Department of Neonatology, University Hospital Zurich, Zurich, Switzerland; ^9^ Institute of Physiology, University of Zurich, Zürich, Switzerland

**Keywords:** congenital heart disease, placenta, brain development, cardiac surgery, neurodevelopmental outcome

## Abstract

**Background:**

Patients with congenital heart disease (CHD) who are operated on after birth are at risk for neurodevelopmental (ND) impairment. Before birth, altered fetal hemodynamics due to the CHD may lead to reduced cerebral perfusion and oxygen supply. The placenta as a critical organ may enhance this pathology.

**Methods:**

Neonates with operated complex CHD were included. We scored the placental pathology and analyzed structural and volumetric brain changes of perioperative brain MRI and ND outcome data using the Bayley III at 1 year of age.

**Results:**

A total of 45 (13 female) patients with D-transposition of the great arteries (*n* = 19, 42.2%), single ventricle CHD (*n* = 14, 31.1%), left ventricular outflow tract CHD (*n* = 7, 15.6%), and other (*n* = 5, 11.1%) were analyzed. Placental findings were abnormal in 21 of 45 patients (46.7%). Pre- and postoperative cMRI were analyzed in 26 (57.8%) and 36 (80%) patients, respectively, while 18 (40%) patients had both (pre-/postoperative) cMRI. Half of our patients had structural brain lesions before (50%) and after (52.8%) surgery, mild intracerebral hemorrhages (pre, 11.1%; post, 22.2%), small cerebral strokes (pre/post, 8.9%), white matter injury (pre/post, 0%/4.5%), and mild hypoxia (pre/post, 4.5%). Abnormal placental findings were not associated with more structural brain lesions but were associated with smaller total brain volumes, cortical gray matter, and cerebellar structures (all *p* < 0.05), but not with ND outcome at 1 year of age.

**Conclusions:**

Abnormal placental findings in patients with complex CHD are associated with smaller brain volumes, underlining the impact of placental function on brain development as a cofactor in patients with CHD.

## 1 Introduction

Neonates undergoing cardiopulmonary bypass surgery for congenital heart disease (CHD) early after birth are known to be at risk for long-term neurodevelopmental (ND) impairments (1, 2). The etiology of the impaired ND outcome is based on multiple pre- and postnatal factors. Before birth, the brain is one of the most rapidly developing organs with a high demand for energy and oxygen and therefore susceptible to impaired development (3). Further prenatal determinants include hemodynamic factors associated with an altered fetal circulation dependent on the type of CHD. For example, in patients with hypoplastic left heart syndrome (HLHS), retrograde aortic flow may lead to reduced cerebral oxygen and nutritional supply (4). These hemodynamic circulatory changes have been described as risk factors for altered fetal brain development due to impaired cerebral perfusion and reduced oxygen and nutritive supply, which might further be enhanced by an associated placental insufficiency (4, 5).

As the placenta is the most critical organ to provide the optimal environment for the development of the fetus, its complex relationship between maternal and fetal factors has recently been defined as the maternal–fetal–placental triad (6, 7). Placental and cardiac development run in parallel, meaning that early placental dysfunction may interfere with cardiac development and vice versa (7). The effect of altered placental development has been shown in transgenic mouse models with trophoblast-specific knockouts leading to lethal types of CHD (8). The proposed specific mechanism for such inference is placental inflammation, which induces a loss of the placental barrier, triggering migration of maternal monocytes capable of altering fetal cardiac structures with the development of CHD (9). On the other hand, the placenta potentially controls, modulates, and compensates for altered heart physiology (10). Therefore, a therapeutic modulation of the placental function within the maternal–fetal environment may become a future treatment option. For that, a better understanding of the relationship between placental pathology and the fetal cardiovascular system is needed (10).

Similar findings of the interdependency of placental function and fetal development, both also determining brain development, have been reported for patients operated for complex CHD during the neonatal period (7), as well as for premature and low birth weight patients with an impaired brain and somatic development and impact on ND outcome (11). Furthermore, it has been found that the combination of premature or low birth weight patients and CHD may both enhance the risk of impaired ND outcome (12).

Based on these findings, we hypothesize that a negative impact of CHD resulting in abnormal placenta findings may be associated with a higher number of structural cerebral lesions, such as cerebral strokes or white matter injuries, reduced brain volumes measured after birth, and an impaired neurodevelopmental outcome at 1 year of age. Therefore, the primary objective of this study was to compare abnormal placental findings and postnatal brain volumes in patients with complex CHD undergoing neonatal cardiopulmonary bypass surgery, and the secondary objective was to evaluate the ND outcome at 1 year of age in relation to placental pathologies.

## 2 Material and methods

### 2.1 Study setting

This study uses data from patients recruited prospectively for two studies, a monocentric (“Heart and Brain,” 2010–2019) and a multicentric study (“BrainCHD,” 2020–2023). Both studies enrolled clinical and neuroimaging data of neonates who were operated on for complex CHD, as detailed in the sections below.

### 2.2 Patients and cardiac diagnosis

We consecutively included full-term neonates undergoing early cardiopulmonary bypass surgery with a complex type of CHD within the first 6 weeks of life. The cardiac diagnosis was grouped according to d-transposition of the great arteries (d-TGA), single ventricle type of CHD, CHD with severe left ventricular outflow tract obstruction (LVOTO), or others. We defined four groups of complexity including biventricular or single ventricle CHD with or without coarctation of the aortic arch, according to the Clancy classification (13). We excluded patients with known genetic comorbidity for this analysis.

### 2.3 Clinical parameters

As perioperative clinical variables, we included gestational age at birth; sex; birth weight (*z*-score); head circumference (*z*-score); Apgar score at 1, 5, and 10 min; and maternal risk factors during pregnancy such as drug abuse (alcohol, nicotine, and others), gestational diabetes, arterial hypertension, and preeclampsia. We defined small or large for gestational age with a birth weight below the 10th or greater than the 90th percentile for gestational age.

### 2.4 Placental findings

Standardized gross examination included the trimmed placental weight (after removal of extraplacental membranes and the umbilical cord), placental size and thickness, macroscopic alterations, features of umbilical cord insertion such as abnormal insertion of umbilical cord at the margin of the placenta or within extraplacental membranes (velamentous insertion), hypercoiling of the cord (>3 coils per 10 cm), or two-vessel cords (only one instead of normally two umbilical arteries). Macroscopic and microscopic placental findings were categorized according to Turowski et al. (6, 14).

### 2.5 Brain MRI

Brain MRI was performed in natural sleep before and after neonatal cardiac surgery before discharge (15). We used a 3.0 T scanner with an eight-channel head coil (Signa HDxt, GE HealthCare, Milwaukee, WI, USA). Structural cerebral MRI findings were systematically assessed by an experienced pediatric neuroradiologist, and diagnoses were categorized into hemorrhage, white matter injury, stroke, signs of hypoxic–ischemic injuries, cerebellar lesions, sinus venous thrombosis, and other lesions (16). To calculate the total brain volume (TBV) and tissue volumes for each patient, T2-weighted images were used. The MR protocol and parameters were published previously (15). T2-weighted images were acquired in three planes: axial, coronal, and sagittal. Images were denoised and bias corrected before super-resolution images were reconstructed using the SVRTK algorithm (17). Next, images were realigned to a reference, and image segmentation was performed using an in-house trained nn-UNet (18). The segmentation followed the annotation protocol of the dHCP pipeline. Automatic segmentation results were visually inspected for quality and corrected manually, if necessary. Volumes were extracted from the segmentation labels as milliliters (ml) using the relevant image statistical modules in the software FSL.

### 2.6 Neurodevelopmental outcome

ND outcome at 1 year of age was determined by examining children with the Bayley Scales of Infant and Toddler Development Third Edition (Bayley III), which was administered by experienced developmental pediatricians blinded to the placental findings. The Bayley III consists of three composite scores, i.e., the cognitive, language, and motor composite scores (mean, 100; SD, 15).

### 2.7 Ethics

Informed consent was obtained from each caregiver, and (2) the study protocol conforms to the ethical guidelines of the 1975 Declaration of Helsinki as reflected in *a priori* approval by the institution's human research committee. For that, both studies have been approved by the responsible cantonal ethical committee in Zürich (Decision numbers: BASEC 2019-01993, KEK StV-23/619/04).

### 2.8 Statistical analysis

Statistical analysis was performed in RStudio (R version 4.3.2). Patient characteristics were described using percentages for categorical variables and mean (standard deviation) or median (interquartile range) as appropriate for continuous variables. To compare patient characteristics and clinical parameters between the two groups, Fisher's exact test for nominal variables and the Mann–Whitney *U* test were used for continuous variables. Placental pathology and brain volume correlation analysis were performed. Brain volume linearly correlates with the neonatal age; therefore, linear models were used to test, whether additional variables such as the placental pathology contribute significantly.

## 3 Results

Placental pathology from 45 patients (13 female) with a complex type of CHD was analyzed. Pre- and postoperative cMRI were analyzed in 26 (57.8%) and 36 (80%) patients, respectively, before and after surgery, and 18 (40%) patients had both pre- and postoperative cMRI. One-year ND outcome was available for 41 of 45 (91.1%) patients, not available due to postoperative death (*n* = 2) or lost to follow-up (*n* = 2).

### 3.1 Patients

The median (IQR) birth weight was 3.300 g (IQR: 3.020–3.680) with a birth weight *z*-score of—0.25 (−1.03 to 0.61). The median gestational age at birth was 39 0/7 weeks (38 3/7–39 6/7). All analyzed patients were born at term, but five term-born infants were small for gestational age. The median head circumference was 34.5 cm (33.8–35.5) with a *z*-score of −0.46 (−1.16 to 0.35). Two patients were microcephalic (*z*-score <1.882), and one was macrocephalic (*z*-score >1.882). The median 1, 5, and 10 min Apgar scores were 8, 8, and 9, respectively. Regarding the maternal comorbidity risk factors during pregnancy, six mothers developed gestational diabetes. No other maternal risk factors were reported (toxins, maternal insulin-dependent diabetes mellitus, arterial hypertension, or preeclampsia).

### 3.2 Cardiac diagnosis

Complex type of CHD included patients with d-TGA (*n* = 19, 42.2%), single ventricle CHD (*n* = 14, 31.1%), severe left ventricular obstruction (*n* = 7, 15.6%), and other CHD (*n* = 5, 11.1%), i.e., pulmonary atresia with ventricular septal defect (*n* = 4, 8.9%) and truncus arteriosus (*n* = 1, 2.2%). According to the Clancy classification, we analyzed 32 (71.1%) patients who underwent biventricular repair of CHD without (*n* = 21, 46.7%) and with aortic arch hypoplasia/coarctation (*n* = 10, 22.2%) and 14 (31.1%) patients who underwent staged single ventricle palliation due to CHD without (*n* = 6, 13.3%) or with aortic arch hypoplasia/coarctation (*n* = 8, 17.8%).

### 3.3 Placental findings

**Figure 1:**
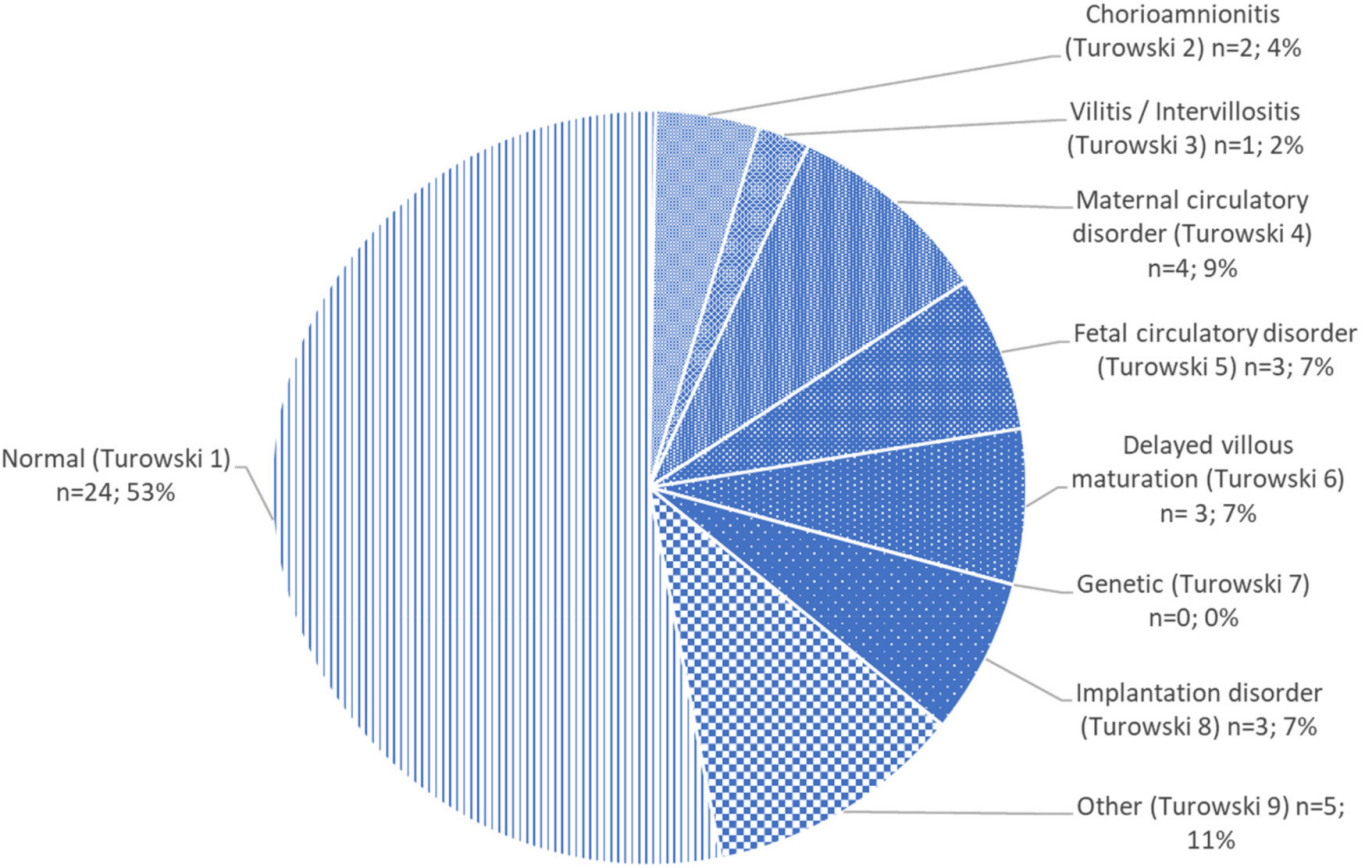
Placental findings in patients with a complex type of CHD.

Figure 1 shows the placental findings according to the Turowski classification system (14) with more detailed characteristics listed in Table 1. Abnormal placental findings were found in 21 (46.7%) patients. Within this heterogenous pattern of abnormal placental findings, maternal and fetal circulatory disorders (Turowski categories 4 and 5) were more frequently found (15.6%, *n* = 7), compared with infectious changes (Turowski categories 2 and 3) (6.7%, *n* = 3).

**Table 1:** Placental findings in patients with a complex type of CHD.

Placental findings	Normal placenta	Abnormal placenta	*p*-value
*N*	24	21	
Placenta weight in g [median (IQR)]	436 (380, 516)	460 (385, 540)	0.657
Placenta birth weight ratio [mean (SD)]	0.13 (0.02)	0.14 (0.02)	0.277
Thickness in cm [median (IQR)]	2.4 (2.0, 3.0)	2.5 (2.0, 2.5)	0.464
Pathologic focal lesion (%)			0.580
No	4 (16.7)	4 (19.0)	
Yes	15 (62.5)	15 (71.4)	
NA	5 (20.8)	2 (9.5)	
Umbilical cord insertion (%)			0.399
Marginal	4 (16.7)	1 (4.8)	
Marginal/velamentous	0 (0.0)	0 (0.0)	
Peripheral	2 (8.3)	2 (9.5)	
Paracentral	14 (58.3)	12 (57.1)	
Central	4 (16.7)	5 (23.8)	
NA	0 (0.0)	1 (4.8)	
Coiling (windings) per 10 cm (%)			0.405
0/10 cm (= UCI <0.1)	2 (8.3)	1 (4.8)	
1/10 cm (= UCI 0.1)	10 (41.7)	4 (19.0)	
2/10 cm (= UCI 0.2)	1 (4.2)	7 (33.4)	
3/10 cm (= UCI 0.3)	8 (33.3)	3 (14.3)	
4/10 cm (= UCI 0.4)	1 (4.2)	2 (9.5)	
NA	2 (8.3)	4 (19.0)	

NA, not available; UCI, umbilical coiling index.

Overall, the median (IQR) placental weight was 444 g (385–536) with a placenta-to-birth weight ratio of 0.136 (0.117–0.150). The placental thickness was measured at 2.5 cm (2.0–3.0). Reduced placental weight below the 3rd percentile was found in three patients and between the 3rd and 5th percentile in three patients, and increased placental weight beyond the 95th percentile was found in one patient.

Overall, umbilical cord insertion was abnormal (defined as marginal, marginal/velamentous, or peripheral) in nine patients (20%), while umbilical cord coiling was abnormal (defined as four coils or more over 10 cm cord distance) in three patients (6.7%). There was no difference in umbilical cord insertion (*p* = 0.399) or umbilical cord coiling (*p* = 0.405) between patients with normal and abnormal placenta according to (14). Placental findings were abnormal in 21 patients (46.7%), showing a heterogenous pattern of maternal and fetal circulatory (15.6%) and infectious findings (6.7%). Pathologic focal findings were found in 15 patients (35.6%).

Furthermore, the number of patients with an increased severity of CHD according to the Clancy classification was not associated with more frequent abnormal placental findings (*p* = 0.253), head circumference (*p* = 0.192), and body weight at birth (*p* = 0.205) (Table 2).

**Table 2:** Comparison of patients with normal and abnormal placental findings at birth including type of CHD.

Patients	Normal placenta	Abnormal placenta	*p*-value
*N*	24	21	
Sex (female)	4 (16.7)	9 (42.9)	0.109
Type of CHD (Clancy classification)			0.253
I (biventricular CHD)	13 (54.2)	8 (38.1)	
II (biventricular CHD + arch obstruction)	6 (25.0)	4 (19.0)	
III (single ventricle CHD)	1 (4.2)	5 (23.8)	
IV (single ventricle CHD + arch obstruction)	4 (16.7)	4 (19.0)	
Gestational age at birth (weeks)	38.99 (0.90)	39.28 (1.33)	0.400
Head circumference at birth (cm)	34.91 (1.41)	34.31 (1.62)	0.192
Head circumference (*z-*score)	−0.19 (1.00)	−0.55 (1.06)	0.252
Body weight at birth (kg)	3.48 (0.64)	3.25 (0.55)	0.205
Body weight at birth (*z-*score)	0.08 (1.34)	−0.43 (1.05)	0.171
Small for gestational age	2 (8.3)	3 (14.3)	0.874

Data are given as *n* (%) or mean (SD).

### 3.4 Brain MRI findings

The pre- and postoperative structural cMRI findings were detected before in 50% and after surgery (52.8%). The most frequently detected abnormality was intracerebral hemorrhages, found in five patients before (11.1%) and in ten patients after (22.2%) surgery, followed by (small) cerebral strokes (before 8.9%, after 8.9%), while white matter injury was only found in one patient after surgery (4.5%), as well as signs of mild hypoxia in one patient before and after surgery (each 4.5%) and other (6.7% vs. 4.5%). As a new postoperative finding, we detected intracerebral bleeding in one patient (4.5%).

Table 3 summarizes the brain volume measurements. We found an association between abnormal placental findings and decreased global and regional brain volumes, including age at scan as a covariate in the model. Nevertheless, the model used has limitations regarding the *R*^2^ values, which were acceptable for TBV, cortical gray matter, and cerebellar structures.

**Table 3:** Total and regional brain volumes in patients with a complex type of CHD and abnormal placental findings.

Predictors	Total brain volume			White matter			Cortical gray matter			Cerebellum			Deep gray matter			Brainstem			Hippocampus		
	Estimates	CI	*p*	Estimates	CI	*p*	Estimates	CI	*p*	Estimates	CI	*p*	Estimates	CI	*p*	Estimates	CI	*p*	Estimates	CI	*p*
*p* (intercept)	−88.42	−206.98 to −30.14	0.141	90.38	51.54 to 129.22	0.001	−121.06	−190.12 to −52.01	0.001	−27.52	−36.43 to −18.61	<0.001	3.60	−7.68 to 14.87	0.525	0.38	−1.86 to 2.62	0.734	−0.84	−2.27 to 0.59	0.245
Placenta group (abnormal)	−40.56	−63.78 to −17.34	0.001	−13.21	−24.54 to −1.88	0.023	−20.53	−30.60 to −10.46	<0.001	−2.61	−4.90 to −0.31	0.027	−2.52	−5.06 to 0.03	0.052	−0.50	−0.95 to −0.05	0.029	−0.39	−0.68 to −0.10	0.010
Age at scan	11.19	8.40 to 13.99	<0.001	1.81	0.91 to 2.72	<0.001	6.48	4.85 to 8.12	<0.001	1.29	1.08 to 1.50	<0.001	0.58	0.31 to 0.85	<0.001	0.15	0.10 to 0.20	<0.001	0.08	0.05 to 0.12	<0.001
Random effects																					
*σ* ^2^	337.97			27.93			180.62			1.56			2.71			0.12			0.05		
*τ*_00_	1,212.24_PID_			333.06_PID_			135.32_PID_			13.30_PID_			15.67_PID_			0.45_PID_			0.20_PID_		
ICC	0.78			0.92			0.43			0.89			0.85			0.79			0.81		
N	45_PID_			45_PID_			45_PID_			45_PID_			45_PID_			45_PID_			45_PID_		
Observations	60			60			60			60			60			60			60		
Marginal *R*^2^/conditional *R*^2^	0.425/0.875			0.142/0.934			0.531/0.732			0.453/0.942			0.160/0.876			0.254/0.845			0.240/0.853		

ICC, intraclass correlation coefficient.

### 3.5 ND outcome at 1 year of age

The median Bayley III composite scores for abnormal or normal placental findings were not statistically different. The median cognitive composite score was 100 (IQR: 95–110) for patients with normal placental findings and 110 (92.5–112.5) for patients with abnormal placental findings. The median language composite score was 91.00 (83.50–99.25) and 97.00 (84.5–104.5), respectively, and the median motor composite score was 88 (82.25–102.25) and 82 (80–91), respectively.

## 4 Discussion

We analyzed the placental findings of neonates with complex CHD undergoing early neonatal cardiopulmonary bypass surgery with a focus on perioperative brain MR findings and ND outcome at 1 year of age. We showed that smaller perioperative total brain volumes, reduced cortical gray matter, and cerebellum were associated with placental pathology.

### 4.1 Placenta findings

Abnormal placental findings were found in nearly half of our patients (46.7%), comparable with the frequency of CHD-associated placental pathology described (19). These frequent histological findings were heterogeneously distributed within the eight groups of the Turowski classification (Figure 1). As expected, we frequently determined abnormal placental findings of maternal (Turowski category 4) and fetal (Turowski category 5) circulatory disorders and inflammatory disease (Turowski categories 2 and 3) (Figure 1). In a recent meta-analysis including a large number of cohort and case–control studies, Spinillo et al. found an association between fetal vascular malperfusion, placental lesions, and brain injury in preterms and term neonates (independent of CHD patients) and impaired ND outcome (20). This was confirmed for neonates with CHD for placental vascular malperfusion within a large case–control study, revealing more patients with chronic maternal and fetal vascular malperfusion lesions than chronic inflammatory lesions. They also reported smaller head circumference at birth in the patients with placental vascular malperfusion (19).

### 4.2 Brain MR structural and volumetric findings

The frequency of perioperative structural cerebral lesions was found to be high in half of the analyzed patients before and after surgery before discharge, but they were not more frequent in patients with abnormal placental findings (21, 22). This might be explained by the circumstance that structural cerebral lesions may rather develop (1) during or immediately after the perinatal transitional period and (2) perioperatively as a consequence of the burden of cardiopulmonary bypass surgery and postoperative intensive care management (23). The time point of the postoperative brain MR before discharge also encounters the effects of postoperative intensive care management.

Nevertheless, we determined smaller total and regional brain volumes after birth in patients with abnormal placental findings (Table 3). Nevertheless, smaller total and regional brain volumes have been described for patients with CHD, even before (24) birth as well as after birth (15). Our study showed smaller regional brain volumes, most pronounced for the cortical gray matter and the cerebellum, both cerebral regions with a higher metabolic energy demand due to the brain volume growth during the third trimester (Table 3), while the higher vulnerability of the white matter might be more associated with apparent structural lesions after birth (25).

In conclusion, placental dysfunction may aggravate the sensitive pre- and perinatal brain development as an additional cumulative factor.

### 4.3 Type of CHD

The distribution of placental abnormalities varied unevenly among the patient groups categorized by the specific type of underlying CHD. We found many single ventricle CHD cases with or without aortic obstruction in the group of abnormal placental findings (21).

While fetal CHD has been described as associated with preeclampsia (26), and low placental weight (27), preeclampsia was not part of our analysis. In contrast, we focused on the potential impact of placental pathology on brain growth until birth and the ND outcome at 1 year of age. While brain growth is impaired in patients with placental pathology, the later clinical impact on ND outcome at 1 year of age was not found. We could not even confirm impaired ND outcome at 1 year of age for other more robust variables such as the type of CHD or the SES, which may be attributed to the multifactorial etiology of impaired ND outcome at 1 year of age.

To our knowledge, this is the first study analyzing the association between placental pathology and 1-year ND outcome in patients with a complex type of CHD. For a comparable risk group of children with hypoxic ischemic encephalopathy, the link between placental pathology and ND outcome could not be determined for acute or chronic placental lesions (28). They only found a correlation between inflammatory placental lesions with higher rate of postnatal epilepsy, which is in contrast to the predominant vascular placental lesion in the CHD population (28).

We also found a high number of low placenta-to-weight ratios below the third percentile, as defined by Almog et al. (29), which was comparable with the findings of Rychik et al. (30). Rychik et al. determined that neonates with TGA as the most prominent CHD diagnosis were associated with placental abnormalities, i.e., placenta-to-weight ratio (30). While lower placenta-to-weight ratio was also described for patients representing a moderate type of CHD, i.e., tetralogy of Fallot, double outlet right ventricle, and large ventricular septal defect, which were not part of our cohort, because have been operated later during infancy, the severity of CHD may not be clearly linked to placental pathology in population-based nationwide study (27). In contrast, in severe types of CHD such as HLHS, unbalanced pro- and anti-angiogenic factors in maternal and fetal cord blood samples have been reported, demonstrating the complexity of vascular placental development and the potential link to the fetal cardiovascular development (31).

Multiple altered vasculogenic pathways may interfere with both the development of CHD and the risk of preeclempsia as a common pathway besides other mechanisms of endothelial dysfunction, impaired implantation, and placental insufficiency (32). Abnormal placental cord insertion and hypercoiling have been shown to be associated with CHD (22, 33, 34); a comparable frequency was found in our cohort (20%). Besides these factors, other factors such as fetal cerebral blood flow pattern and cerebral perfusion as well as other genetic factors may interfere with the fetal brain growth and brain development (4).

A detailed analysis of the impact of maternal gestational risk factor for patients with HLHS undergoing Norwood stage I procedure showed a higher risk of death after stage I due to an impaired maternal–placental–fetal environment (defined as maternal risk factors such as maternal gestation hypertension, preeclampsia, gestational diabetes, and maternal smoking during pregnancy) (35). Therefore, maternal lifestyle, maternal gestational illness, and abnormal placental function may contribute to increased postoperative stage I mortality.

Furthermore, maternal mental health has been shown to influence the maternal–placental–fetal environment resulting in altered prenatal and postnatal risk of maternal psychological distress, anxiety, depression, prematurity, fetal growth restriction, preeclampsia, placental abruption, and neonatal mortality, as well as impaired brain development and long-term impaired ND outcome (36). These studies demonstrate complex pathogenetic pathways including endocrine (i.e., cortisol-mediated and norepinephrine-mediated) including the hypothalamic–pituitary–adrenal axis and inflammatory (cytokine-mediated) mechanisms, which might be modifiable by optimizing fetal and maternal environments in the future.

### 4.4 Brain MR findings and ND outcome

Cerebral white matter lesions have been determined as one of the most predictive MRI markers for impaired adverse developmental outcome in a large European cohort (37). Nevertheless, the presence and type of cerebral lesions in perioperative CHD were not linked with abnormal placental findings, recently shown by Nijman et al. (22). Of note, the abnormal placental findings rather seem to correlate with smaller total and regional brain volume (Table 3) (22). We found the largest impact on smaller total brain volume, smaller cortical gray matter, and the cerebellum, which is in close line with the findings of Nijman et al. (22). Nevertheless, an association between smaller perioperative early postnatal brain volumes and the ND outcome at 1 year of age was not confirmed. This is surprising, because a number of studies showed smaller postoperative brain volumes after neonatal cardiac surgery to be associated with 1-year ND outcome (38), as well as with impaired motoric ND outcome in critical CHD (39), or altered cognitive outcome (40) without including the impact of abnormal placental findings.

Besides placental findings as one potential cofactor for brain growth, a recent review on MRI studies of brain size and growth demonstrated that smaller brain volumes in patients with CHD have been found starting from the fetal period until young adulthood (24, 38, 41–43). This results in an overall growth restriction of all brain regions and is followed by an increase in cerebrospinal fluid volume (41, 44). Furthermore, the association of neuroimaging results beyond reduced brain volumes such as structural MR findings and functional brain changes may generally be linked with poorer neurodevelopmental outcome (45).

### 4.5 Study limitations

The study design was primarily conducted as a clinical descriptive cohort study of patients undergoing early neonatal cardiac surgery with perioperative cerebral MRI and a 1-year ND follow-up study, which was secondarily extended by including all patients with available placental findings. This concept ruled out a case–control study and limited the number of complete patient data sets. The 1-year ND follow-up may be too early to determine significant changes related to the placental pathology and therefore long-term ND assessment may be necessary. Nevertheless, the Bayley III by itself has limitations for ND outcome measurements at 1 year of age (46, 47). Therefore, school-age longitudinal studies on more long-term ND outcomes are needed to determine the clinical impact of placental insufficiency in children and young adolescents at older age. The etiology of placental insufficiency is also multifactorial. Due to the secondary extension of this analysis, we could not describe other factors influencing placental function such as fetal Doppler flow data measurements. Furthermore, we did not perform a detailed analysis of the different abnormal placental findings including immune-histological evaluation. The model used has limitations regarding the *R*^2^ values, which were acceptable for TBV, cortical gray matter, and cerebellar structures. Overall, our patient groups might have been limited and underpowered to reach statistical significance.

### 4.6 Conclusions

Our analysis determined an association between abnormal placental histology and smaller postnatal total and regional brain volumes determined by brain MRI before and after neonatal cardiac surgery, but not with a higher rate of structural brain abnormalities or with an impaired ND outcome at 1 year of age.

The maternal–fetal environment is controlled and modulated by the placenta. The development of the placenta and the embryonic cardiovascular system runs in parallel. Therefore, abnormal fetal cardiovascular development leading to CHD may be accompanied by placental pathology. This relationship may have the potential to provide therapeutic modulations to optimize brain development and ND outcome in this high-risk group of patients with CHD.

Larger clinical case–control studies are needed to confirm the impact of this single factor of the placental pathology besides other modifiable or non-modifiable factors.

In the future, advanced diagnostic imaging tools such as ultrasonography and magnetic resonance imaging of the placenta have to be developed. The therapeutic modulation of the placental function may include its support or replacement, which is needed for optimizing oxygen and nutritional support in children at risk such as CHD fetuses and preterm or low birth weight infants in the future. This may include an appropriate animal model to explore the options of treating placental inflammation and therefore restoring placental barrier function and preventing the development of CHD (9).

## Data Availability

The datasets presented in this article are not readily available due to ethical restrictions. Requests to access the datasets should be directed to walter.knirsch@kispi.uzh.ch.
